# First detection of VEB-1 extended-spectrum β-lactamase-producing *Escherichia coli* clinical isolate in Japan

**DOI:** 10.1128/spectrum.00523-24

**Published:** 2024-09-17

**Authors:** Junko Shindou, Wataru Hayashi, Shizuo Kayama, Liansheng Yu, Hui Zuo, Yo Sugawara, Motoyuki Sugai

**Affiliations:** 1 Department of Laboratory Medicine, Tokyo Metropolitan Tama Medical Center, Tokyo, Japan; 2 Antimicrobial Resistance Research Center, National Institute of Infectious Diseases, Tokyo, Japan; Universidad de Buenos Aires, Buenos Aires, Argentina

**Keywords:** *Escherichia coli*, ESBL, VEB-1, ST95, integron, ExPEC, UPEC, plasmid

## Abstract

The extended-spectrum β-lactamase (ESBL) gene, *bla*_VEB-1_, was identified for the first time in an *Escherichia coli* clinical isolate, JARB-RN-0061, from blood cultures in a Japanese general hospital in 2021. The isolate exhibited high resistance to broad-spectrum cephalosporins, including ceftazidime (MIC >128 mg/L) and cefepime (MIC = 16 mg/L). *bla*_VEB-1_ was identified during whole-genome sequencing and characterization of the isolate. JARB-RN-0061 belonged to the B2-O2:K1:H7-ST95-*fimH*41 lineage and was classified as presumptive extraintestinal pathogenic *E. coli* (ExPEC) and uropathogenic *E. coli* (UPEC). Moreover, the strain harbored multiple virulence genes on the chromosome. The Col156/IncFIB(AP001918)/IncFII(29)-type plasmid (114,216 bp), with *clbB* and *tcpC* genes involved in bacteremia, was unique to the *fimH*41 subclone. The *bla*_VEB-1_ gene was located on a non-typeable and non-conjugative plasmid, pJARB-RN-0061_VEB-1 (17,093 bp). It was embedded in the class 1 integron In1883-like, with multidrug resistance gene cassettes for *aacA4*, *aadB*, *cmlA5*, *qnrVC4*, and *dfrA14*. Notably, comparative analysis of the complete sequence of plasmid pJARB-RN-0061_VEB-1 revealed that it was highly homologous to the *bla*_VEB-1_-harboring plasmid, pMS2H7VEB-1 (100% coverage and 99.99% identity), except for the Tn3 family transposon (4,931 bp) and the plasmid pRHBSTW-00138_5 (97% coverage and 100% identity) harbored by *Klebsiella quasipneumoniae* subsp. *similipneumoniae* strains from hospital sewage in Japan and wastewater influent in the United Kingdom, respectively. The emergence of a human pathogenic *E. coli* clinical isolate with the *bla*_VEB-1_-carrying plasmid in the B2-ST95 worldwide pandemic lineage, characterized by the virulence potential of ExPEC or UPEC but a low prevalence of antimicrobial resistance, would raise public health concerns.

**IMPORTANCE:**

ESBLs are plasmid-mediated enzymes that confer resistance to clinically significant antimicrobial agents, such as broad-spectrum cephalosporins. Recently, the rapid spread of CTX-M-type ESBL-producing *E. coli* has become a global issue, including in Japan, where ESBL production in human pathogenic *E. coli*, such as the ExPEC and UPEC lineages, which typically harbor several virulence genes, is a severe public health concern. To date, VEB (Vietnamese extended-spectrum β-lactamase) producers have been found only in hospital wastewater and rivers in Japan. Thus, we describe the first detection of a very rare human-derived blaVEB-1 gene in the *E. coli* B2-ST95 pandemic clonal lineage that is highly associated with ExPEC and UPEC in a Japanese clinical setting. Furthermore, we characterized the genomic features of plasmids harboring the class 1 integron-borne blaVEB-1. Our findings highlight the significance of closely monitoring ESBL-producing *E. coli* isolates to prevent the potential dissemination of this resistance determinant in Japan.

## OBSERVATION

The global dissemination of extended-spectrum β-lactamase (ESBL)-producing *Enterobacterales*, especially *Escherichia coli*, is a growing concern in clinical setting, as they exhibit resistance to broad-spectrum cephalosporins and other clinically significant antimicrobial agents including fluoroquinolone (1). The Japan Nosocomial Infections Surveillance annual report 2021 indicated a high cefotaxime resistance rate (26.8%) among *E. coli* isolates from inpatients (2). CTX-M-type ESBLs have been disseminated worldwide since the 2000s, and recently, CTX-M-27 and CTX-M-14 producers have become the predominant ESBL-producing *E. coli* in Japan (3, 4). As with other ESBLs that confer high-level resistance to cephalosporins, VEB-1 (Vietnamese extended-spectrum β-lactamase) was initially identified in the pus of a Vietnamese child in 1996 (5). Since its initial discovery, VEB-1 ESBL has been widely distributed among diverse gram-negative rods, including *Enterobacterales*, *Pseudomonas aeruginosa*, and *Acinetobacter baumannii* isolated from clinical samples (5, 6). The dissemination of the *bla*_VEB-1_ gene among bacterial strains has been linked to the transferable plasmid or class 1 integron. Moreover, the acquisition of this gene confers resistance to non-β-lactam antimicrobial agents, including quinolones (5–8). To date, there have been reports of nationwide outbreaks involving the VEB-1-producers from various European and Asian countries (6, 7, 9, 10).

The *E. coli* strain JARB-RN-0061 was recovered from blood cultures drawn from an 89-year-old patient admitted to a general hospital in Tokyo, Japan, in 2021. The antimicrobial susceptibility was determined using the Neg MIC EN 2J and the Neg MIC 3.31E panels (Beckman Coulter Microscan, Brea, CA, USA) and interpreted according to the Clinical and Laboratory Standards Institute document, M100-ED31. The strain exhibited high resistance to broad-spectrum cephalosporins, including ceftazidime (MIC >128 mg/L) and cefepime (MIC = 16 mg/L), and intermediate resistance to levofloxacin (MIC = 1 mg/L). However, it was susceptible to aminoglycosides and fosfomycin (Table S1). The strain was speculated to be an ESBL producer based on the reduction in the MICs of amoxicillin, cefotaxime, and ceftazidime in the presence of clavulanic acid and the weakly-positive double-disc synergy test (Fig. S1). However, multiplex real-time PCR assay using the BD MAX system (Nippon Becton Dickinson, Tokyo, Japan) was negative for the ESBL genes *bla*_CTX-M_, *bla*_TEM_, and *bla*_SHV_.

JARB-RN-0061 was subjected to short- and long-read whole-genome sequencing using the Illumina Hiseq X Five platform (Illumina Inc., San Diego, CA, USA) and GridION (Oxford Nanopore Technologies, Oxford, UK) to comprehend the mechanism of β-lactam resistance. Subsequently, the strain was *de novo* assembled using Unicycler v0.4.8 (11). Genome annotation was performed using the DDBJ Fast Annotation and Submission Tool v1.2.16 (12). The complete genome was analyzed using MLST 2.0, SerotypeFinder 2.0, and FimTyper 1.0 (Center for Genomic Epidemiology [CGE] tools; http://www.genomicepidemiology.org) available for *E. coli* MLST according to the Achtman scheme, O:H serotyping and *fimH* typing, and ClermonTyping v21.03 (13) for *E. coli* phylotyping. Antimicrobial resistance genes, virulence genes, and plasmid replicon types were identified using ResFinder 4.1, VirulenceFinder 2.0, and PlasmidFinder 2.1, respectively, which are also available from the CGE. In addition, the MOB-typer tool available from MOB-suite software v3.1.0 (14) was used to predict the conjugative transferability of plasmids based on the presence/absence of conjugative relaxase, the origin of transfer (*oriT*) sequence, and mating pair formation (Mpf) genes within the plasmid sequences.

**Fig 1:**
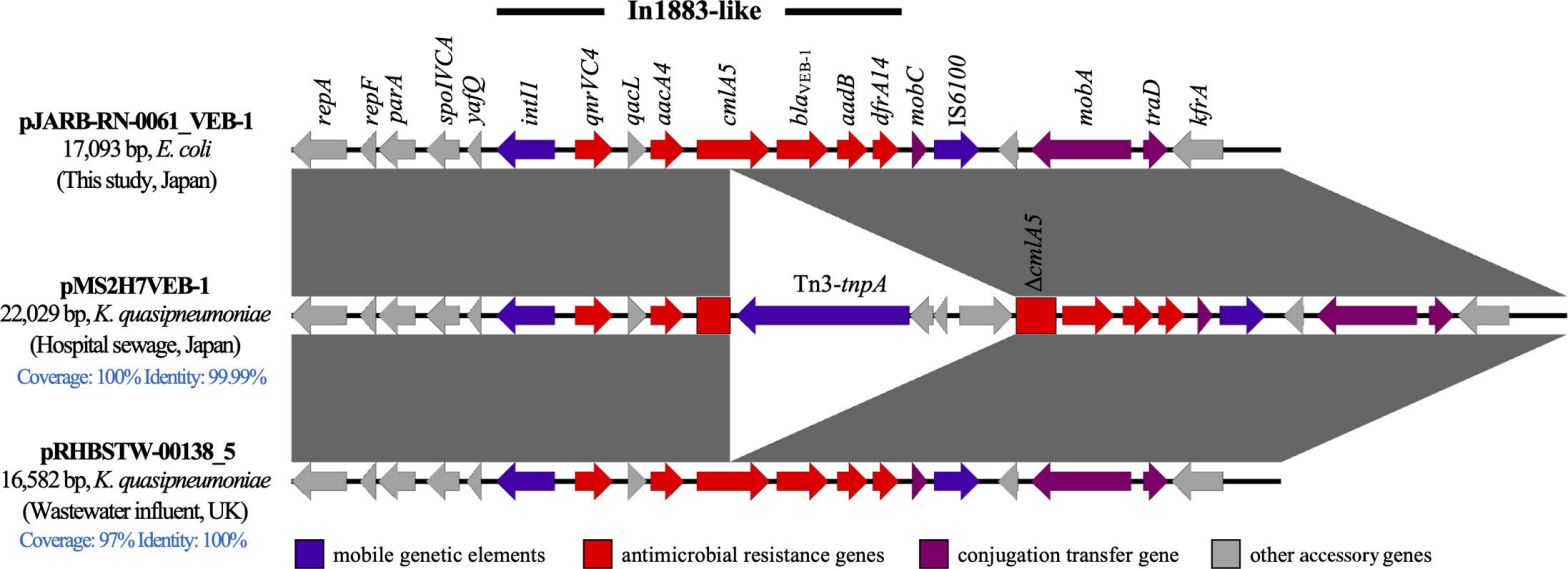
Comparative structures of *bla*_VEB-1_-harboring plasmid pJARB-RN-0061_VEB-1 in this study with plasmids pMS2H7VEB-1 (GenBank accession number LC542613) and pRHBSTW-00138_5 (GenBank accession number CP058135). The arrows show the direction of the predicted coding genes, and the antimicrobial resistance genes are represented as red arrows. The figure was generated using BLASTn and Easyfig (http://mjsull.github.io/Easyfig/).

The whole-genome sequencing revealed a single circular chromosome of 5,094,823 bp and five complete circular plasmids ranging from 1,549 to 114,216 bp. The genome contained 5,049 protein-coding, 22 rRNA, and 92 tRNA genes, with an average G + C content of 50.7%. JARB-RN-0061 harbored acquired antimicrobial resistance genes conferring resistance to β-lactams (*bla*_VEB-1_), aminoglycosides (*aacA4* and *aadB*), phenicols (*cmlA5*), quinolones (*qnrVC4*), and trimethoprim (*dfrA14*), all located on a non-typeable plasmid (17,093 bp), designated pJARB-RN-0061_VEB-1. pJARB-RN-0061_VEB-1 harbored the class 1 integron In1883-like (*intI1-qnrVC4-qacL-aacA4*-cmlA5-*bla*_VEB-1_-*aadB-dfrA14*), the conjugation gene *traD*, and the mobilization genes, *mobA* and *mobC*. Notably, pJARB-RN-0061_VEB-1 demonstrated high homology with the *bla*_VEB-1_-harboring plasmid pMS2H7VEB-1 (LC542613; 100% coverage and 99.99% identity), except for the Tn3 family transposon (4,931 bp), and plasmid pRHBSTW-00138_5 (CP058135; 97% coverage and 100% identity) harbored by *Klebsiella quasipneumoniae* subsp. *similipneumoniae* strains isolated from hospital sewage in Japan and wastewater influent in the United Kingdom, respectively (Fig. 1). Conjugation assays were performed using the *E. coli* J53 (*F^−^met pro* Azi^r^) as the recipient strain. No transconjugants were obtained under laboratory conditions. However, the *E. coli* DH5α transformant acquiring pJARB-RN-0061_VEB-1 exhibited MICs similar to those of the parental strain, including broad-spectrum cephalosporin and levofloxacin (Table S1). pJARB-RN-0061_VEB-1 carried *mobA*, classified into MOBQ family relaxase and *traD* encoding type IV coupling protein as conjugative elements, but missed an *oriT* sequence and Mpf genes and thus was categorized by MOB-typer as “mobilizable” plasmid and not as “self-transmissible (conjugative).”

**Fig 2:**
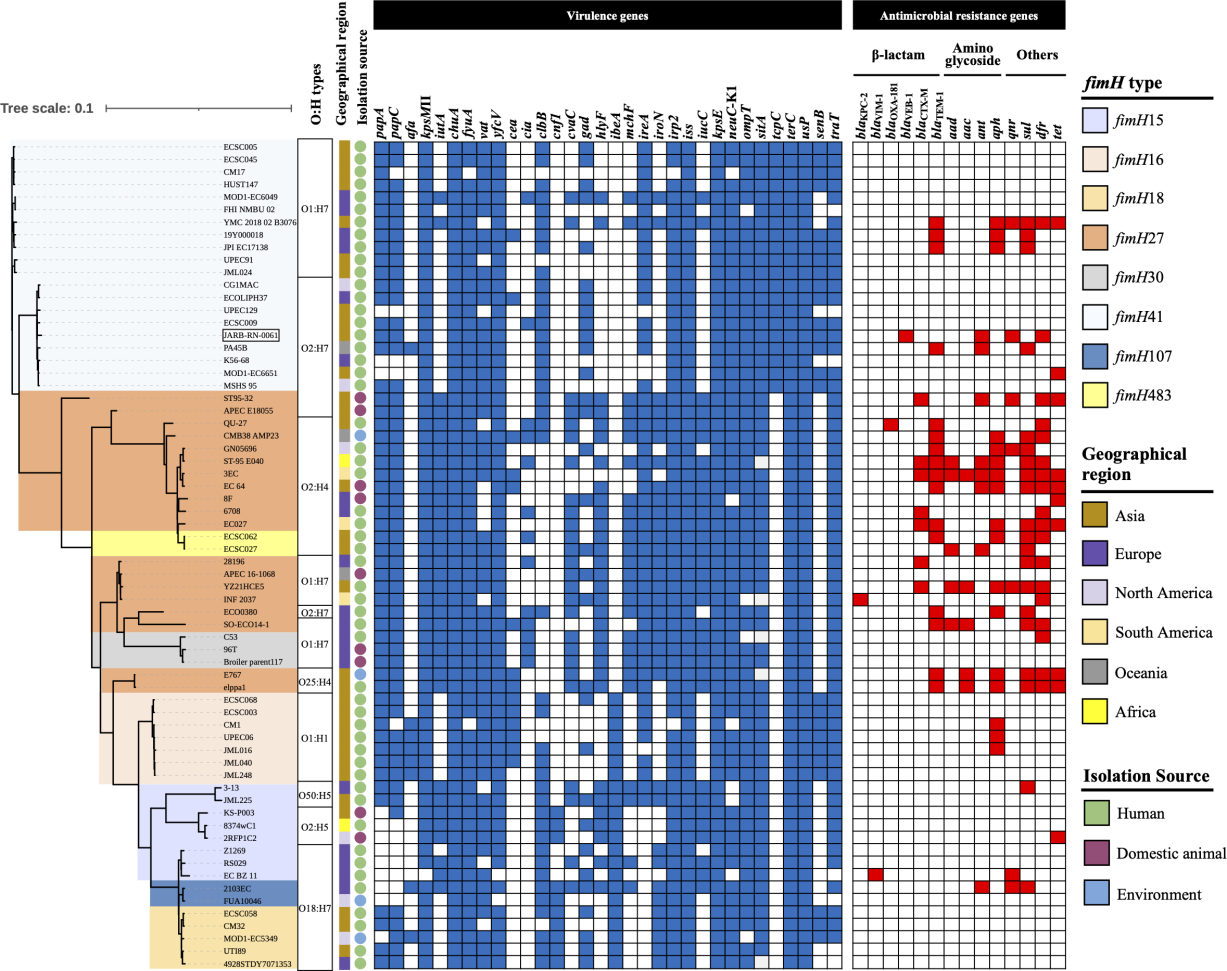
A core-genome SNP-based phylogenetic tree showing the phylogenetic relationships between the VEB-1-producing *E. coli* strain JARB-RN-0061 in this study and 65 *E. coli* B2-ST95 isolated from humans, domestic animals, and environmental samples worldwide. These strains were obtained from the NCBI database accessed in July 2024. The tree was visualized using Interactive Tree of Life (iTOL) v.6 (http://itol.embl.de/). The strain JARB-RN-0061 in the phylogenetic tree is surrounded by a square. The presence (blue and red) or absence (white) of virulence genes and antimicrobial resistance genes predicted by VirulenceFinder 2.0 and ResFinder 4.1 is also shown.

JARB-RN-0061 belonged to the B2-O2:H7-ST95-*fimH*41 lineage, possessed multiple virulence genes on its chromosome and plasmid (Table 1), and was classified as a presumptive extraintestinal pathogenic *E. coli* (ExPEC) and uropathogenic *E. coli* (UPEC) according to the criteria of Johnson et al. (15) based on its contents. Additionally, JARB-RN-0061 was positive for *neuC*, which encodes the K1 capsular antigen, a leading cause of meningitis (16). The phylogenetic tree, which was generated using Parsnp v1.5.6 (17) and based on core-genome single nucleotide polymorphisms (SNPs), included representative genomes of 65 *E. coli* B2-ST95 strains from humans, domestic animals, and the environmental samples in six geographical regions retrieved from the GenBank database (Table S2). These strains were classified into eight distinct *fimH* subclones: *fimH*15, *fimH*16, *fimH*18, *fimH*27, *fimH*30, *fimH*41, *fimH*107, and *fimH*483. This classification was independent of the geographical region or source from which each strain was isolated (Fig. 2). The *fimH*41 cluster, which is further divided into two subclusters consisting of serotypes O1:H7 and O2:H7 with strain JARB-RN-0061, consisted exclusively of human-derived strains, although the geographical region from which they were isolated was diverse. Moreover, the *fimH*41 cluster exhibits a higher prevalence of the polyketide megasynthase gene, *clbB*, and the toll/interleukin 1 receptor-containing protein gene, *tcpC*, both involved in bacteremia (18, 19). In contrast, these genes were rarely found in other clusters. The *senB* gene encoding an enterotoxin was found in *fimH*16, *fimH*18, and *fimH*41 clusters, which was harbored by JARB-RN-0061 and was located on a Col156/IncFIB(AP001918)/IncFII(29)-type plasmid (114,216 bp). The role of a plasmid-mediated *senB* gene as a urovirulence factor in UPEC causing cystitis, pyelonephritis, and urosepsis is known (20). Additionally, the analysis of antimicrobial resistance genes in 66 *E. coli* B2-ST95 strains indicated a relatively low overall prevalence of acquired antimicrobial resistance among the B2-ST95 clones. In contrast, the *fimH*27 cluster demonstrated a notable accumulation of some β-lactamase (carbapenemase and ESBL) and aminoglycoside-modifying genes.

**TABLE 1:** Genetic characteristics of VEB-1-producing *E. coli* strain JARB-RN-0061

Strain		*E. coli* strain JARB-RN-0061
Phylogroup		B2
Sequence type		ST95
Serotype		O2:K1:H7
*fimH* type		*fimH*41
Antimicrobial resistance genes	Chromosome	ND^*a*^
	Plasmid	*bla*_VEB-1_, *aacA4*, *aadB*, *cmlA5*, *qnrVC4*, *dfrA14*
Virulence resistance genes^*b*^	Chromosome	*papA^c^*, *papC^c^*, *kpsM*II*^c^*, *chuA^d^*, *fyuA^d^*, *vat^d^*, *yfcV^d^*, *clbB*, *gad*, *ireA*, *irp2*, *iss*, *kpsE*, *neuC*, *ompT*, *sitA*, *tcpC*, *terC*, *usp*
	Plasmid	*senB*, *traT*
Plasmid replicon types		IncFIB(AP001918), IncFII(29), IncB/O/K/Z, Col156, Col8282, Col(MG828), ColRNAI

^a^ Not detected.

^b^ *papA*, major pilin subunit; *papC*, outer membrane usher P fimbriae; *kpsM*II, polysialic acid transport protein; *chuA*, outer membrane hemin receptor; *fyuA*, siderophore receptor; *vat*, vacuolating autotransporter toxin; *yfcV*, fimbrial protein; *clbB*, polyketide megasynthase; *ireA*, siderophore receptor; *irp2*, yersiniabactin biosynthetic protein; *iss*, increased serum survival; *kpsE*, capsule transport protein; *neuC*, polysialic acid capsule biosynthesis protein; *ompT*, outer membrane protease; *sitA*, iron transport protein; *tcpC*, Toll/interleukin 1 receptor-containing protein; *terC*, tellurium ion resistance protein; *usp*, uropathogenic-specific protein; *senB*, plasmid-encoded enterotoxin; *traT*, outer membrane protein.

^c^ ExPEC-associated virulence genes (15).

^d^ UPEC-associated virulence genes (15).

*E. coli* B2-ST95 is a globally prevalent pandemic clone, highly associated with ExPEC/UPEC strains, known to be pathogenic to both humans and food animals (21, 22) and generally exhibits lower antimicrobial resistance rates than other ExPEC/UPEC strains belonging to STs, such as ST131, ST69, and ST73 (23, 24). ST95 clones are predominant among healthy Japanese individuals and younger patients with community-acquired acute urinary tract infections and are the most common among ExPEC strains recovered from river water in Japan (23, 25, 26). Furthermore, they were phylogenetically related to ST95 clones of human origin.

To our knowledge, VEB producers are yet to be identified in human-derived strains in Japan. However, there have been two reports on VEB producers from aquatic environments: VEB-1-producing *Klebsiella quasipneumoniae* subsp. *similipneumoniae* isolated from hospital wastewater and VEB-3-producing *Aeromonas hydrophila* from a local river (27, 28). The infection source remains unknown as the direct relationship between the patient, water environment, and travel history remains unclear. Class 1 integrons, as mobile genetic elements, have contributed to the distribution and spread of antimicrobial resistance genes at the intra- and interspecific levels among clinical gram-negative bacteria (29), indicating that the gene cassette structure identified in this study may allow the horizontal acquisition of multidrug resistance.

This is the first report characterizing the genomic features of a rare VEB-1-producing *E. coli* isolate from a Japanese clinical setting. The emergence of a human pathogenic *E. coli* clinical isolate harboring a plasmid-borne class 1 integron containing multidrug resistance gene cassettes in addition to *bla*_VEB-1_ raises public health concerns and highlights the significance of close monitoring of ESBL-producing *E. coli* isolates to prevent the potential dissemination of this resistance determinant in Japan.

## Data Availability

The genome sequence data for *E. coli* strain JARB-RN-0061 have been deposited in the DDBJ/EMBL/GenBank database under the BioProject accession number PRJDB15190 and BioSample accession number SAMD00576249. The raw sequence data were deposited in the DDBJ Sequence Read Archive (DRA) under the accession number DRA015598. The complete nucleotide sequence of the plasmid pJARB-RN-0061_VEB-1 harboring the *bla*_VEB-1_ gene has also been deposited in the DDBJ/EMBL/GenBank nucleotide sequence database under the accession number LC745731.

## SUPPLEMENTAL MATERIAL

Tables S1 spectrum.00523-24-s0001.docx Minimum inhibitory concentrations (MICs) for antimicrobial agents.

Tables S2 spectrum.00523-24-s0002.docx Reference genomes of 65 *E. coli* B2-ST95 strains isolated worldwide.

Figure S1 spectrum.00523-24-s0003.pdf Double disc synergy test of *E. coli* strain.

ASM does not own the copyrights to Supplemental Material that may be linked to, or accessed through, an article. The authors have granted ASM a non-exclusive, world-wide license to publish the Supplemental Material files. Please contact the corresponding author directly for reuse.
