# Supplementary material for: First detection of VEB-1 extended-spectrum β-lactamase-producing Escherichia coli clinical isolate in Japan
Source: Microbiol Spectr. 2024 Sep 17;12(11):e00523-24. doi: 10.1128/spectrum.00523-24 (PMC11537020; doi:10.1128/spectrum.00523-24)
Supplement: Tables S1 — Minimum inhibitory concentrations (MICs) for antimicrobial agents. [file spectrum.00523-24-s0001.docx]

Table S1. Minimum inhibitory concentrations (MICs) for antimicrobial agents for VEB-1-producing *E. coli* strain JARB-RN-0061 and *E. coli* DH5α transformant.

| Antimicrobial agents | MICs (mg/L) | | | |
| --- | --- | --- | --- | --- |
|  | *E. coli* JARB-RN-0061 | *E. coli* DH5α transformant  (pJARB-RN-0061_VEB-1) | *E. coli* DH5α |  |
| Ampicillin | >16 | >16 | ≤4 |  |
| Ampicillin/Sulbactam | ≤4/2 | ≤4/2 | ≤4/2 |  |
| Piperacillin | ≤16 | 64 | ≤16 |  |
| Amoxicillin/Clavulanic acid | ≤8/4 | ≤8/4 | ≤8/4 |  |
| Cefoperazone/Sulbactam | ≤8/4 | ≤8/4 | ≤8/4 |  |
| Cefazolin | 8 | >16 | ≤1 |  |
| Cefaclor | 16 | >16 | ≤8 |  |
| Cefpodoxime | 64 | >64 | ≤0.5 |  |
| Cefditoren | >2 | >2 | ≤1 |  |
| Cefotaxime | 64 | 32 | ≤0.5 |  |
| Cefotaxime/Clavulanic acid | ≤0.12/4 | ≤0.12/4 | ≤0.12/4 |  |
| Ceftazidime | >128 | >128 | ≤0.5 |  |
| Ceftazidime/Clavulanic acid | ≤0.12/4 | ≤0.12/4 | ≤0.12/4 |  |
| Ceftriaxone | 64 | 32 | ≤0.5 |  |
| Cefepime | 16 | >16 | ≤1 |  |
| Cefmetazole | ≤0.5 | ≤0.5 | ≤0.5 |  |
| Cefoxitin | ≤2 | 4 | ≤2 |  |
| Cefotetan | ≤1 | ≤1 | ≤1 |  |
| Flomoxef | ≤8 | ≤8 | ≤8 |  |
| Aztreonam | 64 | >64 | ≤0.5 |  |
| Ertapenem | ≤0.25 | ≤0.25 | ≤0.25 |  |
| Imipenem/Cilastatin | ≤0.5 | ≤0.5 | ≤0.5 |  |
| Meropenem | ≤0.25 | ≤0.25 | ≤0.25 |  |
| Gentamicin | ≤2 | 4 | ≤2 |  |
| Amikacin | ≤8 | ≤8 | ≤8 |  |
| Minocycline | ≤2 | ≤2 | ≤2 |  |
| Levofloxacin | 1 | 1 | ≤0.12 |  |
| Ciprofloxacin | ≤0.5 | ≤0.5 | ≤0.5 |  |
| Fosfomycin | ≤4 | ≤4 | ≤4 |  |
| Trimethoprim-sulfamethoxazole | ≤2/38 | ≤2/38 | ≤2/38 |  |
| Tigecycline | ≤0.5 | ≤0.5 | ≤0.5 |  |
