# Supplementary material for: First detection of VEB-1 extended-spectrum β-lactamase-producing Escherichia coli clinical isolate in Japan
Source: Microbiol Spectr. 2024 Sep 17;12(11):e00523-24. doi: 10.1128/spectrum.00523-24 (PMC11537020; doi:10.1128/spectrum.00523-24)
Supplement: Tables S2 — Reference genomes of 65 E. coli B2-ST95 strains isolated worldwide. [file spectrum.00523-24-s0002.docx]

Table S2. Reference genomes of 65 *E. coli* B2-ST95 strains isolated worldwide obtained from NCBI assembly database accessed in July 2024 in this study.

| **Strain** | **Isolation source** | **Specimen** | **Year** | **Geographical region** | **Country** | **BioSample number** | **Sequence number** |
| --- | --- | --- | --- | --- | --- | --- | --- |
| KS-P003 | Bos taurus | feces | 2013 | Asia | Japan | SAMD00077465 | GCA_005391925.1 |
| UPEC06 | Human | urine | 2011 | Asia | Japan | SAMD00077720 | GCA_005397525.1 |
| UPEC91 | Human | urine | 2011 | Asia | Japan | SAMD00077729 | GCA_005397705.1 |
| CM1 | Human | urine | 2006 | Asia | Japan | SAMD00076975 | GCA_005382025.1 |
| CM17 | Human | urine | 2006 | Asia | Japan | SAMD00076973 | GCA_005381985.1 |
| CM32 | Human | urine | 2006 | Asia | Japan | SAMD00076978 | GCA_005382085.1 |
| UPEC129 | Human | urine | 2011 | Asia | Japan | SAMN16443699 | GCA_017356665.1 |
| ECSC003 | Human | blood | 2007 | Asia | Japan | SAMD00076981 | GCA_005382145.1 |
| ECSC005 | Human | blood | 2008 | Asia | Japan | SAMD00076983 | GCA_005382185.1 |
| ECSC009 | Human | blood | 2006 | Asia | Japan | SAMD00076986 | GCA_005382245.1 |
| ECSC027 | Human | blood | 2008 | Asia | Japan | SAMD00077001 | GCA_005382585.1 |
| ECSC045 | Human | blood | 2003 | Asia | Japan | SAMD00077018 | GCA_005382965.1 |
| ECSC058 | Human | blood | 2005 | Asia | Japan | SAMD00077028 | GCA_005383165.1 |
| ECSC062 | Human | blood | 2008 | Asia | Japan | SAMD00077031 | GCA_005383225.1 |
| ECSC068 | Human | blood | 2008 | Asia | Japan | SAMD00077037 | GCA_005383345.1 |
| HUST147 | Human | feces | 2008 | Asia | Japan | SAMD00077083 | GCA_005384265.1 |
| JML016 | Human | feces | 2015 | Asia | Japan | SAMD00077122 | GCA_005385045.1 |
| JML024 | Human | feces | 2015 | Asia | Japan | SAMD00077130 | GCA_005385205.1 |
| JML040 | Human | feces | 2015 | Asia | Japan | SAMD00077143 | GCA_005385465.1 |
| JML225 | Human | feces | 2015 | Asia | Japan | SAMD00077301 | GCA_005388665.1 |
| JML248 | Human | feces | 2015 | Asia | Japan | SAMD00077318 | GCA_005389005.1 |
| YZ21HCE5 | Human | feces | 2021 | Asia | China | SAMN26934416 | GCA_022761445.1 |
| ST95-32 | Chicken | - | 2017 | Asia | China | SAMN12757101 | GCA_008632595.1 |
| E767 | Wastewater | - | 2010 | Asia | China | SAMN11313037 | GCA_004801785.1 |
| YMC_2018_02_B3076 | Human | blood | 2018 | Asia | South Korea | SAMN12854681 | GCA_032647465.1 |
| APEC_E18055 | Chicken | liver | 2018 | Asia | South Korea | SAMN35028029 | GCA_030347115.1 |
| UTI89 | Human | urine | 2018 | Asia | India | SAMN16339622 | GCA_014930875.1 |
| elppa1 | Human | blood | 2019 | Asia | Taiwan | SAMN21169124 | GCA_021655655.1 |
| EC_64 | Chicken | - | 2016 | Asia | Pakistan | SAMN10926076 | GCA_004283235.1 |
| QU-27 | Human | urine | 2019 | Asia | Qatar | SAMN17320627 | GCA_016810015.1 |
| MOD1-EC6651 | Human | feces | 1991 | Asia | Saudi Arabia | SAMN04992481 | GCA_002484905.1 |
| PA45B | Human | urine | 2010 | Oceania | Australia | SAMN06920354 | GCA_002285755.1 |
| APEC_16-1068 | Chicken | - | 2016 | Oceania | Australia | SAMN23170097 | GCA_028752675.1 |
| CMB38_AMP23 | River | - | 2017 | Oceania | New Zealand | SAMN25209229 | GCA_021726255.1 |
| SO-ECO14-1 | Human | blood | 2014 | Europe | Norway | SAMN30161829 | GCA_026132785.1 |
| MOD1-EC6049 | Human | feces | 2010 | Europe | Norway | SAMN05439463 | GCA_002541795.1 |
| 19Y000018 | Human | urine | 2019 | Europe | United Kingdom | SAMN37556623 | GCA_035013245.1 |
| ECO0380 | Human | blood | 2012 | Europe | United Kingdom | SAMEA2204598 | ERS357109 |
| 4928STDY7071353 | Human | feces | 2018 | Europe | United Kingdom | SAMEA104567463 | GCA_902164685.1 |
| 3-13 | Human | feces | 2006 | Europe | Denmark | SAMN08391396 | GCA_002909465.1 |
| Broiler_parent117 | Chicken |  | 2015 | Europe | Denmark | SAMN10980088 | GCA_004960605.1 |
| C53 | Human | urine | 2016 | Europe | France | SAMN14639847 | GCA_012978045.1 |
| 8F | Chicken |  | 2010 | Europe | France | SAMN08391475 | GCA_002911415.1 |
| ECOLIPH37 | Human | feces | 2022 | Europe | Ireland | SAMN38289767 | GCA_033949735.1 |
| K56-68 | Human | urine | 2007 | Europe | Sweden | SAMN08095552 | GCA_002810185.1 |
| RS029 | Human | blood | 2014 | Europe | Sweden | SAMN16393471 | GCA_015163155.1 |
| 6708 | Human | urine | 2017 | Europe | Spain | SAMN20300443 | GCA_019509185.1 |
| JPI_EC17138 | Human | blood | 2016 | Europe | Spain | SAMN22610093 | GCA_022131305.1 |
| 2103EC | Human | feces | 2022 | Europe | Germany | SAMN37903017 | GCA_034721795.1 |
| 96T | Chicken | - | 2010 | Europe | Germany | SAMN08391476 | GCA_002911395.1 |
| 28196 | Human | urine | 2017 | Europe | Russia | SAMN34369800 | GCA_029911685.1 |
| EC_BZ_11 | Human | blood | 2015 | Europe | Italy | SAMN30707467 | GCA_027213255.1 |
| Z1269 | Human | feces | 2015 | Europe | Switzerland | SAMN17073782 | GCA_016433165.1 |
| FHI_NMBU_02 | Human | feces | 2012 | Europe | Norway | SAMN13841230 | GCA_015687605.1 |
| CG1MAC | Human | feces | 2011 | North America | USA | SAMN09386318 | GCA_003258455.1 |
| GN05696 | Human | blood | 2013 | North America | USA | SAMN04393286 | GCA_001621665.2 |
| 2RFP1C2 | Bos taurus | - | 2022 | North America | USA | SAMN31874035 | GCA_026626525.1 |
| MOD1-EC5349 | Wastewater | - | 2003 | North America | USA | SAMN06555267 | GCA_002517335.1 |
| MSHS_95 | Human | urine | 2005 | North America | Canada | SAMN13927616 | SRR10970065 |
| FUA10046 | Wastewater | - | 2015 | North America | Canada | SAMN33741569 | GCA_029335575.1 |
| EC027 | Human | blood | 2005 | South America | Brazil | SAMN15534103 | GCA_025698005.1 |
| 3EC | Human | urine | 2012 | South America | Colombia | SAMN16793237 | GCA_015693785.1 |
| INF_2037 | Human | blood | 2017 | South America | Argentina | SAMN23168963 | GCA_024385835.1 |
| ST-95_E040 | Human | urine | 2013 | Africa | South Africa | SAMN06106836 | GCA_002416845.1 |
| 8374wC1 | Human | feces | 2019 | Africa | Tanzania | SAMN13515575 | GCA_009882835.1 |
