# Supplementary material for: First detection of VEB-1 extended-spectrum β-lactamase-producing Escherichia coli clinical isolate in Japan
Source: Microbiol Spectr. 2024 Sep 17;12(11):e00523-24. doi: 10.1128/spectrum.00523-24 (PMC11537020; doi:10.1128/spectrum.00523-24)
Supplement: Figure S1 — Double disc synergy test of E. coli strain. [file spectrum.00523-24-s0003.pdf]

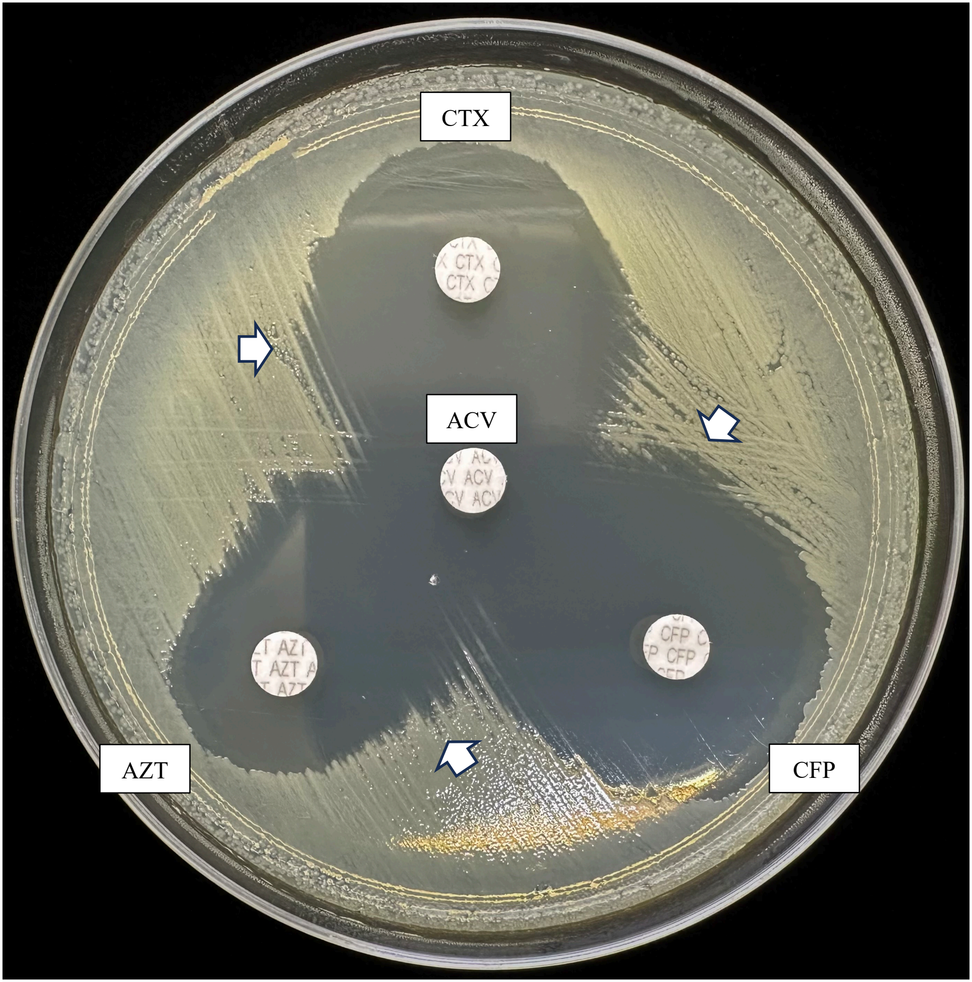

Fig. S1. Double disc synergy test of *E. coli* strain JARB-RN-0061 using cefotaxime (CTX, 30 µg), cefepime (CFP, 30 µg) and aztreonam (AZT, 30 µg) discs along with amoxicillin/clavulanic acid disc (ACV, 20 µg/10 µg) in the center. White arrows indicate the enhancement of zone inhibition around CTX, CFP and AZT discs.
